# Prof. Gross

**Published:** 1850-11

**Authors:** 


*Prof. Gross.*—Prof. Gross passed through this city, recently, on his route to the city of New York, to commence his professorial labors in the Chair hitherto occupied by Dr. Mott. No one can doubt the ability of Prof. G. to sustain himself in his new position, and none who know him can wish his shadow less than it is.

